# The early development of the onychopod cladoceran *Bythotrephes longimanus* (Crustacea, Branchiopoda)

**DOI:** 10.1186/1742-9994-11-10

**Published:** 2014-02-07

**Authors:** Frederike Alwes, Gerhard Scholtz

**Affiliations:** 1 Humboldt-Universität zu Berlin, Institut für Biologie/Vergleichende Zoologie, Philippstr. 13, 10115 Berlin, Germany; 2 Current address: Institut de Génomique Fonctionnelle de Lyon (IGFL), 32-34 avenue Tony Garnier, Lyon 69007, France

**Keywords:** Cell lineage, Cladocera, Crustacea, Development, Cleavage pattern

## Abstract

**Introduction:**

Within arthropods, several crustacean groups are unique in their early development due to their stereotyped cell division patterns and cell lineages. However, it is still unclear whether these cell division patterns are homologous between the various crustacean groups and whether they could indicate the ground pattern of Tetraconata (Crustacea and Hexapoda). In this study we describe the early development of the raptorial water flea *Bythotrephes longimanus* as a representative of the Cladocera within branchiopods.

**Results:**

In *B. longimanus* the early cell lineage and the cell division pattern are stereotyped up to the fifth cell division cycle. As a morphological marker a nurse cell remnant (**ncr**) identifies the cell lineage of the smallest and division delayed blastomere up to the 16-cell stage. This marker might be indicative of the germ line. By combining histology, confocal laser scanning microscopy, and 4D microscopy, we reconstruct the early cell lineage and cell division pattern and follow transient formations of cell morphological structures in their temporal and spatial behavior up to gastrulation.

**Conclusions:**

Correspondences to the early cleavage pattern of other Cladocera suggest that the described pattern can be assumed to be ancestral for either the entire Cladocera or for the majority of the Cladocera comprising Anomopoda, Ctenopoda and Onychopoda. The comparison to the cell division patterns of other crustacean groups such as Malacostraca, Ostracoda, and Copepoda reveals similarities that allow for a discussion of a common pattern for the crustacean groups and a ground pattern for the Tetraconata.

## Introduction

In contrast to other arthropods, crustaceans display a highly diverse early development. For instance, cleavage modes range from holoblastic to superficial over mixed cleavage, in which holoblastic cleavage turns into superficial cleavage and vice versa. This is even true within the major crustacean groups such as Malacostraca or Branchiopoda [1]. This renders the reconstruction of the ancestral mode of crustacean development a difficult task despite several comparative accounts [2-4]. Moreover, the search for the ancestral cleavage pattern in crustaceans has not only been hampered by the great developmental divergence between and within crustacean taxa but by uncertainties concerning the phylogenetic relationships of the group. Recently, the view that paraphyletic crustaceans form a clade together with Hexapoda, which is called Tetraconata or Pancrustacea, has become dominant [5-7]. However, it remains elusive which crustacean taxon is the sister group to the hexapods [5-7]. Despite this uncertainty, it is obvious that the reconstruction of the early development of ancestral crustaceans has to include hexapod embryology and refers in fact to the tetraconate stem species.

In addition to other variable aspects of early cleavage, a stereotyped cleavage pattern occurs in a number of crustacean groups, e.g. within Malacostraca (Amphipoda: [8-10]; Euphausiacea: [11,12]; Dendrobranchiata: [13-15]), within Branchiopoda (Cladocera: [16,17]; Anostraca: [18,19]), and in Cirripedia [20-23], and Copepoda [24]. Until the late 1990′s the prevailing idea was that Arthropoda and the spirally cleaving Annelida are closely related, forming the Articulata (see [25]). This view implied the interpretation of stereotyped crustacean cleavage as modified spiral (e.g. [3,11,26], but see [27-30]). The current interpretation of Arthropoda being more closely related to the Cycloneuralia forming Ecdysozoa renders the assumption of crustacean cleavage as spiral obsolete. It has instead provoked the resumption of studies on the early cell lineage pattern of other holoblastically cleaving arthropod groups like pycnogonids [31]. However, since no stereotyped cleavage pattern is detectable in pycnogonids or other arthropods apart from crustaceans, there is the question as to whether the various examples of stereotyped cleavage in crustaceans follow a corresponding pattern and whether stereotypy of cleavage evolved once in the tetraconate lineage or independently in the various crustacean lineages.

The establishment of techniques like 4D microscopy for cell lineage tracing and 3D reconstructions of histological slides or CLSM images allows us to follow and illustrate temporal and spatial events more easily. Therefore, we are able to provide more morphological developmental data for ground pattern reconstructions even within crustacean groups.

Using these recent methods in this study, we describe the early cleavage pattern and the temporal and spatial appearance of cellular substructures of *Bythotrephes longimanus* Leydig, 1860 as representative of cercopagididan Onychopoda within Cladocera (water fleas). So far only one onychopod species, the polyphemid *Polyphemus pediculus*, has been studied a century ago with respect to its early development [16,17]. Our findings permit the detailed comparison with the results of this previous study resulting in the reconstruction of the putative ground pattern of onychopod early development. Furthermore, we discuss the different cleavage types described for cladocerans (holoblastic, superficial, and mixed) with respect to stereotypic cleavage pattern and address possible links between cell fate restriction, such as the germ line, and cell morphological markers. In a comparative approach we discuss possible phylogenetic implications of the described cleavage pattern for either subgroups of the Cladocera or the Cladocera as a whole, reconstructing the plesiomorphic cladoceran cleavage pattern.

Overall similarities of the holoblastic cleavage of Malacostraca, Phyllopoda, and free living Copepoda have been recognized earlier [11,17,24]. However, focusing on the cleavage with respect to the cell lineage and subsequent gastrulation processes, Gerberding and Patel [32] oppose similarities between Malacostraca and the remaining groups. To contribute to the question of to what extent it is possible to describe a common early cleavage pattern for Tetraconata, we explore further pattern similarities to other crustacean groups.

## Results

### General characteristics of the *Bythotrephes longimanus* development

**Figure 1:**
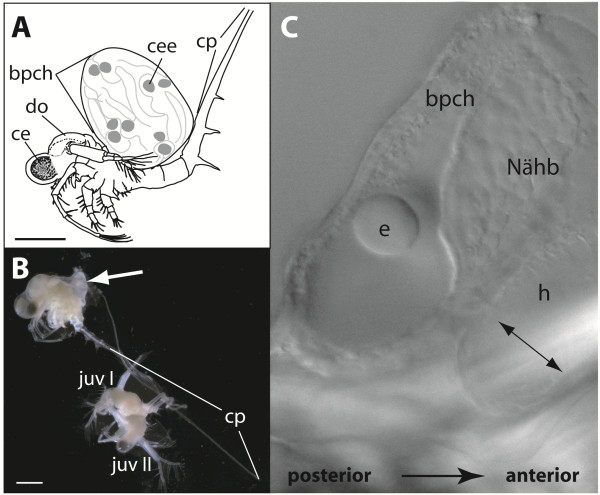
***B. longimanus *females carry their embryos in a dorsal brood pouch until they release them as juveniles. A** Schematic drawing of a female of *B. longimanus* carrying embryos in its dorsal brood pouch (bpch). The degree of pigmentation of the embryonic compound eyes (cee) is visible through the cuticle of the brood pouch. From late gastrulation on the embryos increase in size, and depending on how many embryos the brood pouch is carrying, it can eventually swell up to several millimeters. **B** Female with its two hatched juveniles (juv I, II) still attached with their caudal processes wrapped around the caudal process of the mother. The regenerated brood pouch with new eggs is already visible (white arrow). Animals fixed in Bouin. **C** Brood pouch containing eggs that have just been released. The pumping heart (h) causes a movement of the early eggs (e) in the liquid within the brood pouch (bpch). ce - compound eye (adult), do - dorsal organ, Nähb – Nährboden (=nurturing tissue), scale bar - 1 mm.

The parthenogenetic reproduction cycles of *B. longimanus* start with the formation of a dorsal brood pouch in which the embryos develop and hatch as juveniles (Figure 1). Immediately after hatching, a newly formed brood pouch is visible as a small dorsal triangular lobe (Figure 1B).

**Figure 2:**
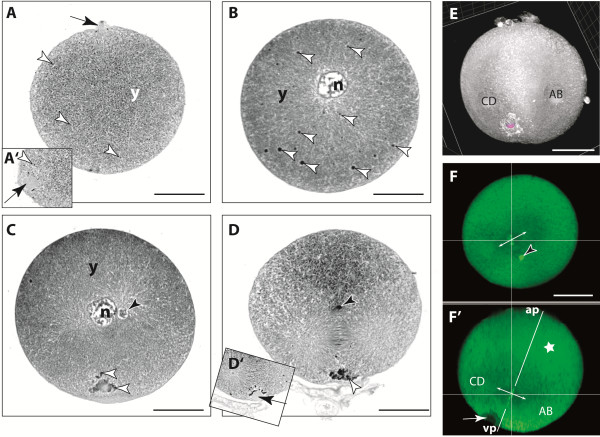
**During the first division structural changes are cell-morphologically visible. A** Zygote with the polar body remnant characterized by relicts of the spindle apparatus (black arrow). The yolk (y) is evenly distributed and fine dense particles are scattered throughout the egg (arrow heads). **A’** Detail of a section close to the surface of the same egg as in **A**. Only some small granules appear around the ‘nurse cell remnant body’(**ncr**) (black arrow). **B** Zygote with central nucleus (n). The dense granules now appear much clearer (white filled arrow heads). **C** Section of a zygote with ‘nuclear blister’ (black filled arrow head). Dense granules (white filled arrow heads) appear around the ncr (not visible). **D** The 1st mitotic division in metaphase. The ds (black filled arrow head) appears at the metaphase plane connected to the spindle apparatus. Dense granules around the **ncr** (not visible) are indicated by the white arrow head. **D’** Detail of the **ncr** (black arrow head) of the same egg as in **D. E** The spatial relationship between the positions of two nuclei and the **ncr** (violet sphere) shifted towards the CD nucleus. **F, F’** Metaphase of the first division. **F** The **ds** (white bordered arrow head) appears at the metaphase plate. **F’** View of the xz axes of the same image stack of **F**. The region around the vegetal pole (asterisk) contains more condensed yolk. The direction of the first mitosis (double arrow) is perpendicular to the a/v-axis (white line) ap - animal pole and vp - vegetal pole. The **ncr** (arrow) is shifted towards the CD nucleus. **A-D, A’, D’** Semi-thin sections stained with toluidin blue. **E** 3D reconstruction based on semi-thin sections (toluidine blue). **F, F’** Sytox-stained confocal image stacks in ‘section mode’ view. Scale bars - 20 μm.

**Figure 3:**
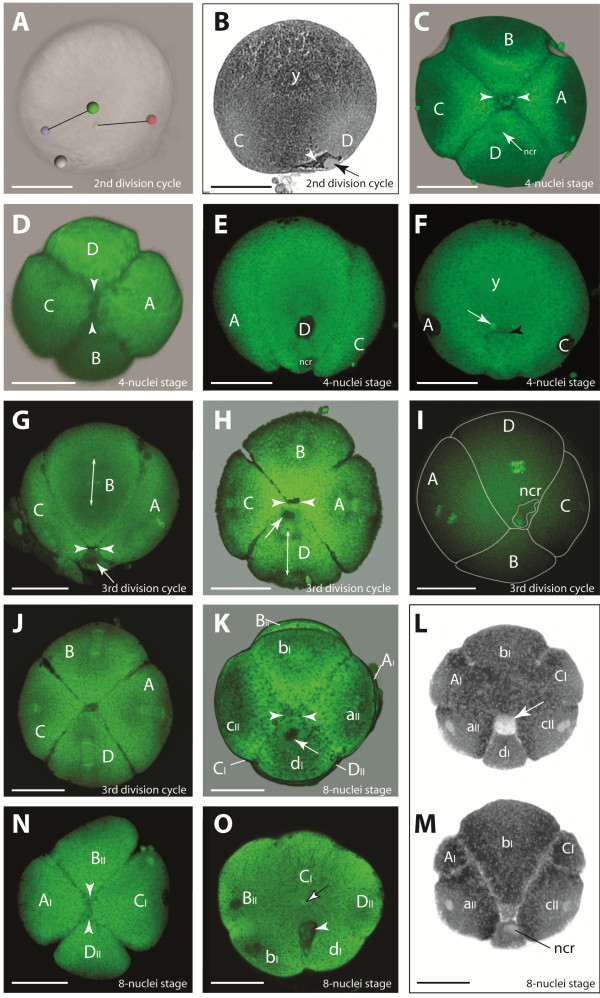
**The 2nd and 3rd division from the 2- to 8-nuclei stage. A** 3D reconstruction using semi-thin sections of the 2nd division (metaphase). Black lines: spindle orientations; A - yellow, B - red, C - green, D – blue: the four quadrants; the gray sphere: **ncr. B** Semi-thin section of the same egg. Dense granules (white arrow head) surround the **ncr** (arrow) in quadrant D. Dense yolk (y) dominates the animal region. **C** The vegetal pole at the 4-nuclei stage. The short cross furrow between B and D (arrow heads) is still covered by the cavity-like structure. **D** The animal pole at the 4-nuclei stage with a cross furrow between A and C (arrow heads). **E** Quadrant D at the 4-nuclei stage, animal pole is facing up. The **ncr** in D is shifted towards C. **F** Confocal section of the same egg as in **E**. The yolk (y) is condensed at the animal half. The white arrow marks the **ds** and the black arrow head the cavity-like structure. **G** The vegetal part of B forming a short cross furrow (arrow heads) with D; white arrow: **ncr. H** The vegetal B-D contact, slightly later as in **G**; white arrow: **ncr. I** The transient delay of the D division. **J** The vegetal pole with the quadrants in late anaphase. **K** Vegetal pole after 3rd division; arrow heads: cross furrow between B and D derivatives. **L, M** Semi-thin section of the same embryo. The membranes do not completely penetrate the yolk **(L)**. The white arrow points to the cavity-like structure between D and B. The **ncr** lies in dI **(M). N** The animal pole of an 8-nuclei stage showing the cross furrow between macromeres aII and cII (arrow heads). **O** Animal-vegetal section of an 8-nuclei stage. The **ds** (black bordered arrow) has reached the center of the egg. Towards the vegetal pole, the cavity-like structure is visible (white arrow head) between B and D micromeres. Scale bars - 20 μm.

**Figure 4:**
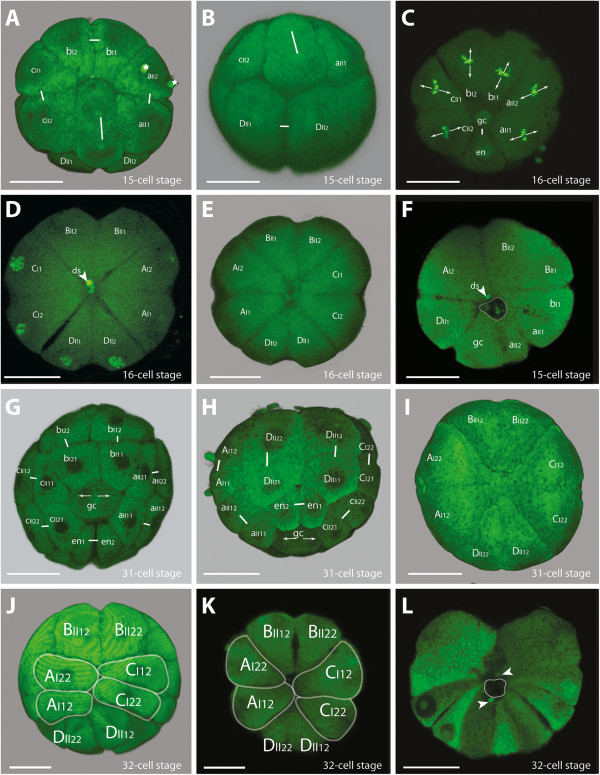
**Embryos of *B. longimanus *during the 4th and 5th division cycle towards the 32-cell stage. A** View of the vegetal pole with the division delayed gc. Micromeres aII, bI, and cII have divided in equatorial direction, while gc divides in animal-vegetal direction (small stars: cellular relicts as artifacts). **B** Embryo at a similar stage as in **A** with view of the D derivatives. The delayed division of gc is at the top (long white line). **C** View of the vegetal pole of a 16-cell stage with all cells at metaphase apart from gc and en. gc is in contact to all micromeres at the vegetal pole: aII1, aII2, bI1, bI2, cII1, cII2, and en. **D** Single image of a confocal scan near the center of the embryo where the macromeres appear in a clover leaf-like arrangement. The **ds** lies in the center. **E** The animal pole at the 16-cell stage. BII1 and BII2 have not contact to DII1 and DII2. **F** Confocal section of the area with the small cavity-like structure in the center (surrounded by a white line). **G** View of the vegetal pole. The division of gc is delayed compared to en, resulting in a 31-cell stage. **H** View of the D derivatives of a similar stage as in **G**. The direction of the division of en and gc is perpendicular to that of DII1 and DII2 (short lines represent the sister cell relationships of all visible cells; in gc the direction of division is marked by arrows). **I-K** View of the animal pole with different positions of macomeres in which BII and DII derivatives do not contact each other. **L** Confocal section with two **ds** (white arrow heads) adjacent to the cavity-like structure (surrounded by a white line) in the center of the embryo. Scale bars - 20 μm.

**Figure 5:**
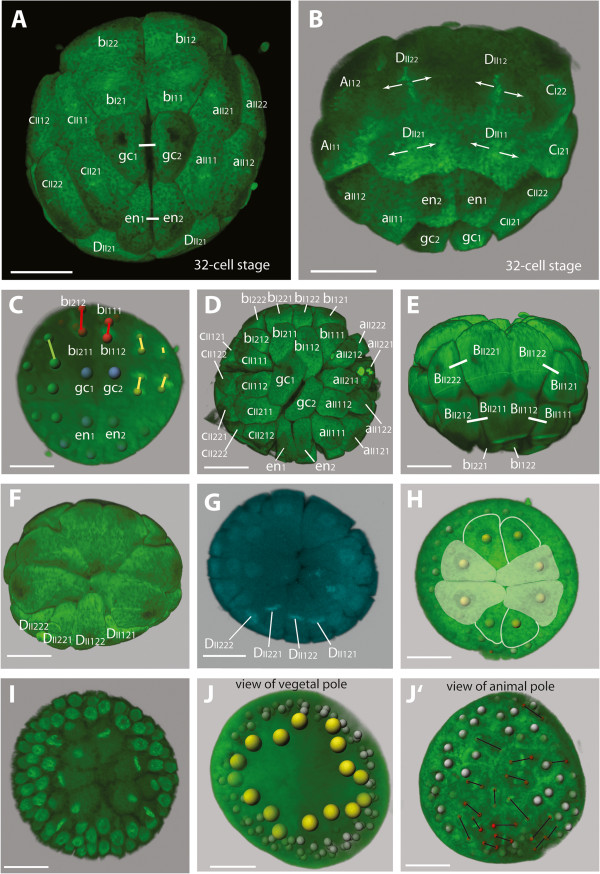
**The processes from the 6th division cycle on through the beginning of gastrulation. A** View of the vegetal pole of the 32-cell stage during 6th division cycle. Apart from gc1, gc2, en1, and en2 the cells are in metaphase. **B** An embryo at a similar stage as that in **A** with view of the D derivatives. The orientation of their divisions is indicated by arrows. **C** Embryo at the 6th division cycle in which bI21 and bI11 (nuclei represented by red spheres) adjacent to gc1 and gc2 divide perpendicularly compared to the direction they reveal in **A. D** 3D reconstruction of the 60-cell stage after the completion of this cycle. The positions of the two cells gc1 and gc2 in this embryo are not symmetrical with respect to the other cells and appear slightly rotated clockwise. **E** 60-cell stage with the view of the B derivatives. **F** View of the animal pole of the same embryo as in **E**; the D derivatives are smaller compared to the other macromeres with DII122 being the largest D macromere. **G** Animal pole of a 60-cell stage, with the d macromeres at metaphase initiating the following division cycle. **H** View of the animal pole of an embryo consisting of 175 cells of which 17 are in mitosis at the vegetal pole. The nuclei of the macromeres are arranged in a circle. Each two AI and CI derivatives (transparent white overlay) are in contact and the BII and DII macromeres do not share a common border. **I** View of the vegetal pole of an embryo comprising 236 cells showing eight macromere derivatives arranged in a ring. **J, J’** Embryo of 215 cells with 16 macromeres arranged star/cross-like and with 21 cells in mitosis at the animal pole (represented by red spheres in **J’**). Scale bars - 20 μm.

Within the next 15–25 minutes two to six subitaneous eggs are released into the posterior part of a newly formed brood pouch. As soon as the eggs enter the brood pouch, they float in the nutrient solution and take a spherical shape (Figures 1C and 2). From the third division cycle on, the eggs can become slightly flattened at the animal and vegetal pole (Figure 3), and during the following cycles, the shape of the eggs can range between strictly spherical to oval-shaped (see Figures 4 and 5). The size of the eggs ranges between 50 and 80 μm even within the same clutch of a female. However, the development of the eggs is synchronous.

**Figure 6:**
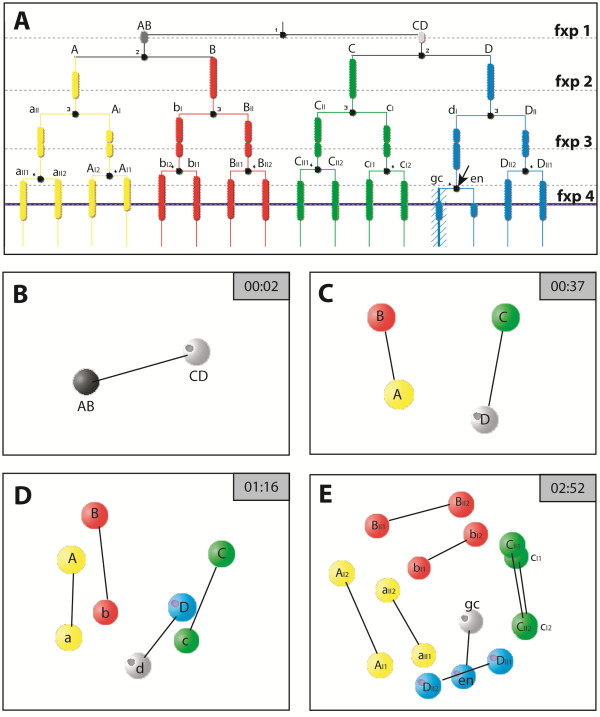
**Cell lineage analysis of the timeline of the first divisions and the spatial positions of the nuclei. A** Cell lineage analysis of *B. longimanus* from the 2-nuclei stage to the 16-cell stage. The four colors represent the quadrants of the 4-nuclei stage, with A - yellow, B - red, C - green, D - blue. The black points mark the time of division. The broken lines show the ‘fixed points’ time line levels of which four are shown as 3D models **(B-E). B-E** Screen shots of the 3D model in SIMI°BioCell at the 4 fixed points (fxp 1 -4) in **A**. The vegetal pole faces down in **B-D**. In **B** to **D** the nucleus associated with the **ncr** is symbolized as gray sphere. In **E** the embryo has slightly rotated so that the view is from the animal pole. The sister cell relationships are indicated by the black connecting lines. en: endoderm cell, gc: germ cell.

**Figure 7:**
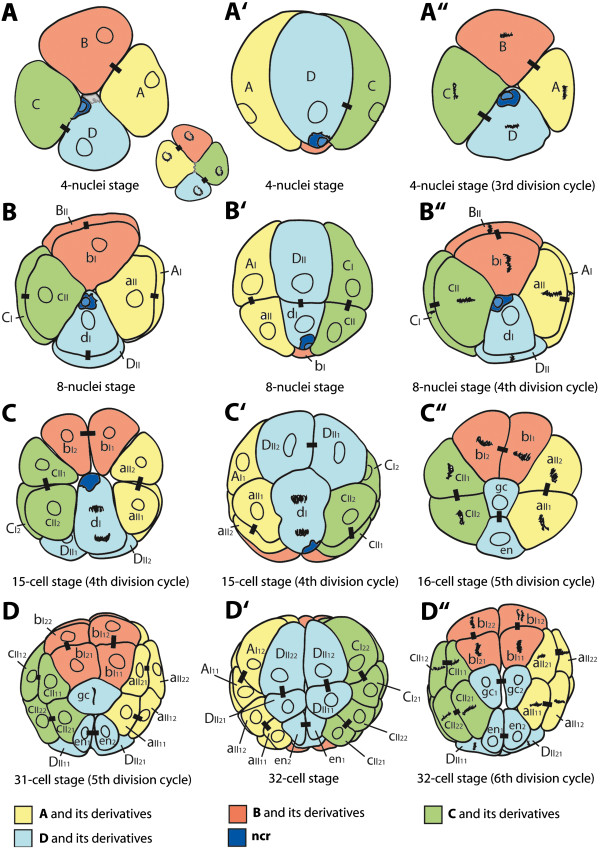
**Schematic overview of the early division pattern of *B. longimanus *until the 32-cell stage.** Lines represent the cleavage furrows as they appear at the surface and do not penetrate the complete egg until the 4th cleavage. Short lines mark the sister cell relationship according to the previous division. The four colors represent the quadrants of the 4-nuclei stage, with A - yellow, B - red, C - green, D - blue. The gray area in D indicates the region that is covered by yolk droplets on the surface of the cell. Left column **(A**-**D)**: View of the vegetal pole (except in **A** in which the view of the animal pole is added). Middle column **(A’**-**D’)**: View of the D derivatives, vegetal pole faces down. Right column **(A”-D”)**: View of the vegetal pole during the division cycle.

**Figure 8:**
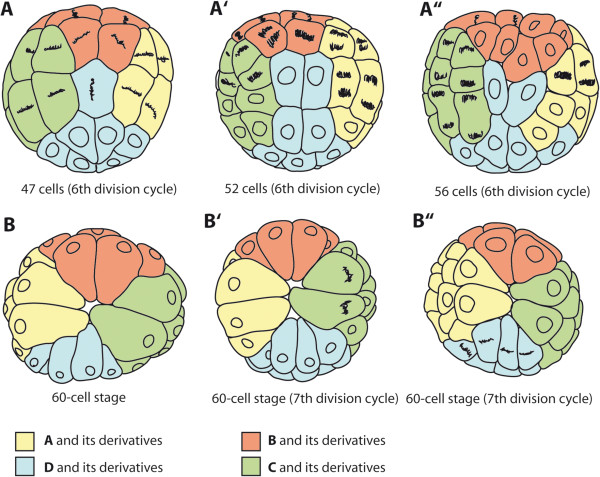
**Schematic overview of the 6th division cycle and the initiation of the 7th division cycle in *B. longimanus*.** The four colors represent the quadrants of the 4-nuclei stage, with A - yellow, B - red, C - green, D - blue. **A-A”** View of the vegetal pole during the 6th division cycle. **A** The division cycle is initiated in A-, B-, and C descendants and at the same time gc has not divided yet. The direction of bI11 and bI12 adjacent to gc are inclined in this embryo (double arrows). **A’** Embryo of 52 cells during the 6th division. **A”** Embryo of 56 cells during the 6th division in which A and C derivatives are delayed in division. **B – B”** view of the animal pole of three different embryos of 60 cells. With **B’** and **B”** already starting the next (7th) division cycle. The 7th cycle can be initiated in different quadrants, as shown here for C derivatives (in **B’**) and for D derivatives (in **B”**).

In the early stages, the eggs reveal very fine and evenly distributed yolk (Figure 2A). They are tightly surrounded by a thin, transparent egg envelope and throughout the subsequent development, the embryos appear nearly transparent. Early cleavages follow a stereotyped pattern (Figures 3, 4, 5, 6, 7, and 8). During the first three division cycles, the embryos undergo an incomplete cytokinesis (Figure 3). Therefore, it is referred to nuclei-stages until the fourth division. During these three cycles, however, the cell membranes increasingly penetrate the complete cell mass, leading to a holoblastic cleavage from the fourth division cycle on (Figures 4 and 5). For a schematic overview of the following description of the cleavage events including the nomenclature, see Figures 7 and 8.

### From the zygote and the 1st mitotic division to the 2-nuclei stage

The zygote of parthenogenetically produced eggs emerges from an automictic division [33] which occurs close to the surface of the egg before or during its release into the brood pouch. This event results into the extrusion of a polar body that is characterized by relicts of the former spindle apparatus (Figure 2A). The polar body remains attached to the egg until the first nuclear division, but can disappear earlier.

The embryonic nucleus moves towards the center of the cell and enlarges to about 10 μm (Figure 2B). Its chromosomes remain condensed in the state of prophase until the next division (Figure 2B, C). At this point, the nucleus is accompanied by a small nuclear unit of about 3 μm in diameter which reveals the same structural organization and seems to be not connected to the main part (Figure 2C). We call this structure ‘nuclear blister’.

Another distinctive cellular structure is a sphere-shaped particle with about 5 μm in diameter. It is evenly stained in toluidine blue stained sections (Figure 2A’ , D’), but remains unstained when using fluorescent nuclear dyes (see Figure 2F’). It is already detected during the preceding maturing division at the periphery of the cell (Figure 2A’) and remains at the periphery until later stages in the region opposite the polar body (see e.g. in Figure 3H, K). In analogy to [17] it is referred to this structure as a nurse cell remnant (**ncr**).

Throughout the yolky cytoplasm, small dark stained granules appear in toluidine blue stained semi-thin sections (Figure 2A, B): in the early embryonic cell they are barely distinguishable as very fine dark spots and evenly distributed throughout the cytoplasm (Figure 2A). They aggregate into larger particles at the time when the nucleus enters the prophase (Figure 2B). At this point the particles are still distributed throughout the cytoplasm, but their size gradually increases when they migrate towards the periphery of the egg in the region of the **ncr** (Figure 2B). Thereafter, these granules are detected exclusively in the region around the **ncr**. Here they gradually appear as a reorganized, more or less compact structure (Figure 2C, D, D’). The region containing the **ncr** is referred to as the ‘vegetal pole’ (Figure 2E, F). The opposite region, with its region containing mainly the yolky cytoplasm, is referred to as the ‘animal pole’ (Figure 2E).

The 1st embryonic mitotic division occurs about 60–75 minutes after the juveniles of the previous reproduction cycle have been released. With the initial formation of the spindle apparatus, the dividing nucleus is shifted towards the vegetal pole (Figure 2F). At the metaphase, the **ncr** lies adjacent to the metaphase plate at the periphery of the egg (Figure 2F’). An imaginary line through the metaphase plate in animal-vegetal direction reveals that the **ncr** is slightly shifted towards one of the two daughter nuclei, which only then can be distinguished as the nucleus **CD** adjacent to the **ncr** and the nucleus **AB** (Figure 2F).

During the metaphase arrangement of the chromosomes, a small compact structure of condensed chromosomes can be distinguished at the level of the metaphase plate towards the center of the egg in both histological staining and Sytox staining (Figure 2D, F). Figure 2D shows this compact structure as an integrated part of the spindle apparatus. It can still be detected at the same position during the 2-nuclei stage. It is reasonable to assume that this is a mitotic transformation of the nuclear blister observed in the prophase nucleus prior to the division. During the following development, no similar additional nuclear unit is found. However, one or two Sytox-positive dense spots (referred to as **ds**) can be found in the embryo throughout early development.

The 1st division is meridional and results in a 2-nuclei stage which is superficially cleaved, but the cell membranes do not separate the eggs into two individual cells (superficial cleavage) (Figure 2E). However, the two equal sized cell regions can be linked to each nucleus. The regions can be distinguished due to the position of the **ncr**, which is always found to be shifted towards the nucleus of the region designated **CD**. The other nucleus with its related cytoplasmic region is **AB** (Figure 2E).

### From the 2nd division cycle to the 4-nuclei stage

The 2nd division occurs synchronously in both nuclei about 40 min after the 1st division. The spindle directions of **AB** and **CD** form a small angle in relation to the animal-vegetal axis and to each other (Figure 3A). The four resulting quadrants with their related nuclei differ in size, the position of the **ncr**, and their position to each other; **CD** gives rise to the quadrants **C** and **D**, while the **D** quadrant is the smallest and contains the **ncr** (Figure 3B). The other nucleus **AB** divides into **A** and **B**, with **B** being the quadrant opposite to **D** (Figure 3C). The **ncr** remains at the periphery of the egg. Again, the cell membranes do not divide the egg into four separate cells, but the membranes reach slightly deeper into the egg center compared to the previous division (Figure 3E, F). The cleavage runs meridionally and perpendicularly to the first cleavage and is slightly unequal (Figure 3C, D).

When the division is completed, the superficial cell membranes of the two quadrants **B** and **D** contact each other forming a short additional ‘plane’ which is referred to as a short ‘cross furrow’. At the animal pole a short cross furrow is formed more clearly by the two quadrants **A** and **C**, running perpendicularly to the plane of the **BD** cross furrow (Figure 3C, D).

The **BD** contact plane is covered by a more or less spherical cytoplasmic region, which has a different droplet-like yolk structure which is different from the rest of the yolk structure and reveals scattered small Sytox stained granules (Figure 3F, I). This cytoplasmic structure is first detected when the nuclei have entered the telophase of the 2nd division cycle. Subsequently, it sinks towards the center during the next two division cycles. It is referred to this structure as a ‘cavity-like structure’. However, its identity or function remains unclear. Close to this cavity-like structure, one or two compact **ds** are found (Figure 2F).

At this stage, the **ncr** in the **D** quadrant is close to the cross furrow, slightly shifted towards the **C** quadrant (Figure 3E). During this division cycle, it becomes apparent that the early divisions of *B. longimanus* occur in two mirror images, in which the arrangement of the four quadrants occurs in two chiral variants. Based on these two chiral variants, the following development also occurs in two mirror images.

Towards the end of the 4-nuclei stage, the nuclei have reached the egg’s surface within the ‘vegetal half’ of the four quadrants (Figure 3F). The ‘animal half’ contains the main part of the yolk-filled mass of the cell quadrants (Figure 3F). The position of the nuclei within the blastomeres towards the vegetal pole leads to an unequal division of the next division cycle.

### From the 3rd division cycle to the 8-nuclei stage

The 3rd division cycle starts about 40 minutes after the previous one. The mitotic spindles of this division are orientated in animal-vegetal direction (Figure 3G, H). They form almost synchronously and only a transient delay of mitosis is detected in **D** during the early anaphase (Figure 3I). During this division cycle, the cytoplasmic cleavage is still not complete and the resulting eight blastomere compartments are still connected in the center (Figure 3L, M, O). This cleavage is perpendicular to the previous divisions and therefore equatorial with respect to the animal-vegetal axis. It is unequal, giving rise to the quartet of the smaller sized **aII**, **bI**, **cII**, and **dI** at the vegetal pole and to the quartet of the macromeres **AI**, **BII**, **CI**, and **DII** at the animal pole (Figure 3J, K, O). Among the micromeres the smallest is **dI** and among the macromeres the smallest quadrant is **DII** (Figure 3N).

The cross furrow at the vegetal pole as an additional contact plane between the **B** and **D** quadrant of the 4-nuclei stage is preserved at the 8-nuclei stage between **bI** and **dI** (Figure 3G, I, K). At the animal pole the cross furrow between the macromeres **AI** and **CI** is less preserved. However, in those embryos showing a clear cross furrow at the animal pole, it is always formed by **AI** and **CI** (Figure 3N). The macromeres **BII** and **DII**, in contrast, never contact each other at the animal pole (Figure 3N). The small cavity-like structure with its small scattered Sytox-positive granules adjacent to the micromere **dI** has moved slightly towards the center (Figure 3F, O). Either one or two **ds** are found in the center of Sytox stained embryos close to this structure (Figure 3O). The nuclei remain at the surface of the egg (Figure 3L, M, O), and the position of the macromere nuclei shift towards the vegetal pole. Figure 6 depicts the positional constellations of the nuclei during the first three cleavages.

### From the 4th division cycle through a 15-cell stage to the 16-cell stage

The division spindles of the **A**, **B**, and **C** derivatives and of macromere **DII** form synchronously 40 minutes after the previous division. They are oriented parallel to the equatorial plane of the embryo, except for the one belonging to **dI** (Figure 4A, B). During this division cycle, **dI** divides in an animal-vegetal direction and is therefore oriented in the same direction as in the previous division cycle (Figure 4A, B). Its division is clearly delayed and a stage with 15 blastomeres is temporarily formed (Figure 4A, B). The resulting daughter cells **gc** and **en** are the smallest micromeres (Figure 4C). During the division of **dI**, the **ncr** is still visible, but after the completion of this division, the **ncr** disappears (compare Figure 4A and G).

The 4th division cycle is the first division in which blastomeres are divided into separate cells (e.g. Figure 4D, F). From now on divisions are holoblastic. At the 16-cell stage, the cavity-like structure is located in the center of the egg (Figure 4F). Towards the animal pole, the eight macromeres meet in the center of the embryo (Figure 4D). At the animal pole, however, the **B** derivatives and **D** derivatives do not contact each other (Figure 4E). The two macromeres **DII1** and **DII2** are smaller compared to the other macromeres, with **DII1** being slightly larger than **DII2** (Figure 4E). At the vegetal pole, **gc** is in contact to all other micromeres, namely **aII1**, **aII2**, **bI1**, **bI2**, **cII1**, **cII2**, and **en** (Figure 4E). The former cross furrow at the vegetal pole described for the 4- and 8-nuclei stage is preserved as the contact plane between **en**, **bII1**, and **bII2** (Figure 4C).

### From the 5th division cycle to the 32-cell stage

As soon as the micromere **dI** has completed the division of the fourth cycle into **en** and **gc**, the fifth division cycle has already started in all remaining cells (Figure 4C). Except for **en** and **gc**, the cells divide synchronously around 40 min after the previous division. The mitotic spindles of the micromeres **aII1**, **aII2**, **bI1**, **bI2**, **cII1**, and **cII2** orient in an animal-vegetal direction, almost radially with respect to the division-delayed micromere **gc** (Figure 4C).

After the initiation of the fifth division cycle in the other cells, the **en** micromere divides into **en1** and **en2**, leading to a transient stage of 31 cells (Figure 4G, H). As soon as **en** is divided, micromere **gc** starts its division into **gc1** and **gc2** (Figure 4G). The resulting 32-cell stage is composed of the four derivatives of **dI** that just have completed their division and the remaining 28 cells that are about to enter the sixth division cycle (see in Figure 5A, B).

At the animal pole, the **D** derivatives **DII1** and **DII2** divide parallel to each other in an animal-vegetal direction, perpendicular to the division of **en** (Figure 4H). Their cleavage is unequal, leading to the larger daughter cells **DII12** and **DII22** towards the animal pole and smaller daughter cells **DII11** and **DII21** towards the vegetal pole. The remaining macromeres **AI1**, **AI2**, **BII1**, **BII2**, **CI1**, and **CI2** also divide unequally, but their division spindles orient at an inclined angle with respect to the animal-vegetal axis. Each two macromeres of the same precursor (e.g. **AI1**, **AI2**) emit their smaller daughter cell towards the vegetal pole between them; because the direction of the division is inclined to each other (see Figure 7).

The **D** macromeres at the animal pole are still smaller compared to the other macromeres (Figure 4I-K). This difference is also distinct among the macromeres **DII12** and **DII22**, with **DII12** being larger than **DII22** (Figure 4I, K). The position and contact planes of **A** and **C** derivatives can be quite different and the embryos in Figure 4J, K exemplify the three most different situations that can occur: either it is two **A** and **C** macromeres in contact, in which case it is always those diagonally opposite each other that are in contact (i.e. the macromeres **A12** and **C12** or **A22** and **C22**) (Figure 4J, K), or the contact line is very short and all the four macromeres seem to meet at the vegetal pole (Figure 4I). Notably, the **B** macromeres (i.e. **BII12** and **BII22**) are never in contact with **DII** derivatives (i.e. **DII12** and **DII22**).

At the vegetal pole of the 32-cell stage, the former cross furrow is maintained by the contact plane of two **b** (**bII21** and **bII11**) and two **d** derivatives (**gc1** and **gc2**), but regarding the sister cell relationships, these cells are now at right angles to each other (Figure 5A). With respect to size, the micromeres do not differ significantly from each other. However, the shape and arrangement of **gc1**, **gc2**, **en1**, and **en2** is specific: after completing the 32-cell stage, the quartet of **gc1**, **gc2**, **en1**, and **en2** reveals the shape of an acute-angled triangle pointing its tip into the center of these four cells (Figure 5A).

### From the 6th division cycle to the 60-cell stage

By the time the division-delayed cells **gc1**, **gc2**, **en1**, and **en2** have completed their mitosis of the fifth division cycle at the 32-cell stage, the remaining 28 cells have already started their sixth division cycle (Figure 5A, B). From this cycle on, the cleavages no longer follow a strict stereotyped pattern. Variation occurs with respect to the orientation of mitotic spindles, to the timing of divisions of single cells, and with respect to the position of single cells among their neighbor cells (cell-cell contacts). However, the division of the **D** derivatives **gc1**, **gc2**, **en1**, and **en2** at the vegetal pole is consistently delayed compared to the timing of divisions of the remaining cells and **gc1**, **gc2**, **en1**, and **en2** remain temporarily undivided, leading to a stage of 60 cells.

The initiation of the sixth cycle occurs in either **A**, **B**, or **C** derivatives, but no regular pattern could be observed (compare with Figures 5C and 8A, A”). The **D** derivatives have never been observed to initiate the 6th division cycle. The highest degree of variability in the division timing is shown in Figure 8A revealing that the 6th division cycle can in some cases be initiated as early as the 31-cell stage, at which the **gc** has not yet divided into **gc1** and **gc2**.

The variability with respect to the direction of division on the opposite pole exclusively concerns the division of **b** micromeres **bI11** and **bI21** adjacent to **gc1** and **gc2**. Either their division is parallel to their former sister cells **bI12** and **bI22** (Figures 5A, D and 8A’) or the direction of the mitosis of **bI11** and **bI21** is oriented at a right angle or inclined to that of **bI12** and **bI22** (Figure 8A’ , A”). All the remaining micromeres consistently divide perpendicularly to the direction of the previous cycle (Figure 5A). This is also true for the eight macromeres **AI12**, **AI22**, **BII12**, **BII22**, **CI12**, **CI22**, **DII12**, and **DII22** at the animal pole, whose direction of mitosis is again inclined with respect to the animal-vegetal axis, but almost perpendicular to the previous division cycle (Figure 5E).

Again the eight macromeres of the 32-cell stage divide unequally. At the animal pole, the **D** derivatives consistently do not contact any **B** macromere derivatives (Figure 5F). The difference in size between the **D** macromeres and the remaining cells is more apparent at the 60-cell stage. Especially the difference in size between the two **D** macromeres **DII122** and **DII222** is preserved by **DII122** being larger than **DII221** (Figure 5F). The range of variance of the blastomere pattern at the animal pole is presented in the scheme in Figure 8A-A”.

### Further development of gastrulation processes and general observations

The 7th division cycle generally begins in the macromeres and their smaller sister cells. Interestingly, the starting blastomeres comprise the derivatives of only one clone, i.e. of either **AI**, **BII**, **CI**, or the **DII** macromeres (Figure 5G). At the vegetal pole **en1** and **en2** divide in an animal-vegetal direction. From this point onwards, the difference in the spindle orientation between different embryos prevents the tracing of single cells. Also the timing of the following division cycles varies in different cells and no regular sequence or cell division pattern could be observed. Therefore, the following two rounds of division do not display stages with a defined number of cells.

During the 8th division, eight macromeres are found at the animal pole (Figure 5H). Their division during this cycle is still unequal, with eight larger macromeres at the animal pole and their smaller sister cells positioned towards the vegetal pole. The nuclei of the macromeres remain arranged in a circle around the animal pole (Figure 5A, C). The unequal division of the macromeres proceeds at least through the 9th division cycle (Figure 5E).

The **ds** in the center of the embryos is visible until the 9th division. Thereafter, during advanced gastrulation, the **ds** disappears.

After the 9th division cycle, the embryo comprises around 450 cells. The division activity decreases by this time and individual cells detach from the surface at the vegetal pole and initiate the gastrulation process by cell immigration. The identity of the immigrating cells remains unclear and the gastrulation process seems to not follow a regular sequence in terms of the timing of spatial arrangements (Figure 5I, J, J’). Therefore, which role the derivatives of **gc** and **en** play during gastrulation and further development must remain open.

## Discussion

### Resting and subitaneous eggs

Most detailed developmental studies of Cladocera go back to the beginning of the 20th century and more recent studies generally do not focus on the cell lineage or peculiarities in cell morphology during the earliest cleavages [34,35]. Cladocerans produce two types of eggs. The resting eggs (‘winter eggs’) emerge from bisexual or gamogenetic reproduction and the subitaneous eggs (‘summer eggs’) emerge from parthenogenetic reproduction. There are only few studies available on the development of the resting eggs of Cladocera [36-40]. All these studies mainly focus on cell morphological characters like yolk distribution or penetration of cell membranes, based on the prevailing interest at that time in whether the cleavage of the resting eggs is holoblastic or not, and although detailed in description, cell lineages of resting eggs have not been traced (e.g. [38]).

The alternation between parthenogenetic and gamogenetic reproduction of cladocerans is derived within branchiopods [41,42]. As a consequence, parthenogenetic reproduction is seen as the derived mode, since sexual reproduction is found in most remaining crustacean taxa. But this does not inevitably mean that the cell lineage and cell division pattern of the asexually produced eggs are also derived. The structural and physical requirements of resting eggs rather argue that several aspects of the resting eggs and their development are derived. In a comparative study of both types of eggs in the ctenopod cladoceran *Holopedium gibberum*, von Baldass [39] concludes that the development of both resting and subitaneous eggs is identical except for the diapause, which is inserted during the development in resting eggs. The following discussion focusses primarily on parthenogenetically produced subitaneous eggs.

### Early development of *Bythotrephes longimanus* reveals distinct similarities to that of another onychopod, *Polyphemus pediculus*

The early cleavages of the *Bythotrephes longimanus* reveal many distinct similarities to those of another onychopod cladoceran, *Polyphemus pediculus*[16,17,43,44]. As *B. longimanus, P. pediculus* reveals a stereotyped early cell division pattern and cell lineage occurring in two mirror images [17]. The first two cleavages are meridional and equal, followed by the equatorial and unequal 3rd cleavage, which results in four micro- and four macromeres. Both species show a mixed cleavage type [45,46] with early superficial cleavage shifting to total cleavage during more advanced development. However, holoblastic cleavage in *P. pediculus* begins with the 5th division cycle [17], whereas in *B. longimanus* it starts with the 4th cleavage division. During the 4th division cycle, the **D** micromere is delayed in division and the orientation of its division is identical in the two species. The following sequence of mitoses, the direction of the divisions, and the size of cells are very similar in both species until the 6th division cycle – during this cycle, the first variability in the direction of divisions in *B. longimanus*, is found, which then leads to a variant pattern in the next cycle. In contrast, the cleavage of *P. pediculus* is stereotyped until at least the 9th division cycle, and except for a slight variance in the sequence of mitosis, the pattern does not vary in *P. pediculus*[17]. Due to this invariance, single cells could be traced until the 9th division cycle and were assigned to their future cell fate [17]: the 7th division cycle leads to an 118-cell stage with 106 ectoderm-, 6 mesoderm-, 4 endoderm-, and 2 germ cell precursors. The germ cell precursors go back to the cell lineage of the **dI** micromere. The fate of the **dI** micromere is reported to be determined from the 1st cleavage on by the existence of a **ncr** at the same location as is found in *B. longimanus*[17]. The 8th division cycle is followed by a 236-cell stage including 212 ectoderm-, 8 endoderm-, 12 mesoderm-, and 4 germ cell precursors. During the 9th division cycle (with 424 ectoderm-, 12 mesoderm-, 16 endoderm- and 8 germ cell precursors) gastrulation occurs and 28 cells at the vegetal pole sink towards the blastocoel. After gastrulation, the embryo comprises around 460 cells [17]. In *B. longimanus* gastrulation occurs at a similar stage and cells are internalized at a stage of approximately 455 cells. However, the internal cells could not be differentiated with respect to their mesoderm, endoderm or germ cell precursor identity. Cercopagidae, to which *B. longimanus* belongs, and Polyphemidae, containing *P. pediculus,* do not form a monophyletic group within Onychopoda. Most analyses show that Polyphemidae is the sister group to the remaining onychopod taxa (e.g. [41]). Hence, the high degree of similarity between the early cleavage patterns of both species indicates that the corresponding cleavage characters are part of the onychopod ground pattern.

### Holoblastic and superficial cleavages and consequences for the cleavage pattern

In general, a holoblastic mode is emphasized with respect to the early determination of Cladocera, since a holoblastic cleavage is often seen as a prerequisite for a determined development [2,47]. However, many examples of cladocerans that are often cited as being holoblastically cleaving and early determined in cell fate begin their development without completely separating the blastomeres into cells; for instance, *Holopedium gibberum*[39]*, Daphnia pulex*[48] and *Polyphemus pediculus*[17], in which total separation is attained only from the 5th cleavage on in *H. gibberum* and *D. pulex*, and from the 4th cleavage on in *P. pediculus*. According to these findings, it seems worthwhile to study the cleavage of *D. magna* in more detail, since there is the claim that it follows the superficial mode [34]. Nevertheless, these examples undergo a stereotyped early division pattern with a ‘cell’ fate determination of ‘blastomeres’ as nuclei with linked areas from the first cleavages on. Therefore, *Bythotrephes longimanus*, *H. gibberum*, *D. pulex,* and *P. pediculus* demonstrate that a stereotyped cell fate determination does not necessarily depend on a complete holoblastic cleavage.

Within the cladocerans, we also find examples that clearly show a superficial cleavage mode; for instance, in *Simocephalus vetulus*[49] and *Leptodora kindtii*[50,51]. The 1st division cycles in *S. vetulus* begin with the formation of energids that first divide in the center of the embryo and later move towards the egg surface, where the nuclei at the surface transform into the blastodermal cells surrounding a central mass of yolk [49]. Nevertheless, during blastoderm formation this species [49] presents a pattern corresponding to what is found in *Moina rectirostris*[52] or *P. pediculus*[17] (both with mixed cleavage) at the vegetal pole prior to gastrulation: 2 or 4 division-delayed cells surrounded by presumptive ectomesoderm cells and at one side neighbored by presumptive mesendodermal cells.

In *L. kindtii*, the early divisions of the energids follow a stereotyped cleavage pattern [50]. However, a pattern similar to what is described for *S. vetulus* prior to gastrulation has not been described [49,50]. The first two divisions in *L. kindtii* result in four nuclei that are somewhat shifted towards one pole [50]. The spindles of the 2nd division are at a slight angle to each other, forming a similar arrangement to the 4-nuclei stage in *B. longimanus*. This shows that division pattern can be invariant regardless of whether the embryos undergo holoblastic, superficial, or mixed modes during the first division cycles.

### The ncr as a morphological cell marker

The nurse cell remnant **ncr** in *Bythotrephes longimanus* corresponds to the structure described in *Polyphemus pediculus* not only with respect to the position in the putative lineage of the **gc**, but also with respect to its histological composition: a small spherical and regularly stained body that is surrounded by dense associated granules revealing no strict shape and an irregular ‘surface’ [17,43]. In both cases the **ncr** decays after the 4th division: in *P. pediculus* the **ncr** first leaves the cell surface and moves towards the center during the 8-cell stage [17]; in *B. longimanus* it remains at the surface. The decay of the **ncr** occurs along with the change from incomplete to holoblastic cleavage in *B. longimanus*. In *P. pediculus*, the transition to holoblastic cleavage occurs one cycle earlier and at the same time the **ncr** already shows first indications of its decay [17].

The temporal connection of these two events may be explained by the necessity of a structural marker close to a determined nucleus prior to the change to complete cytokinesis. After switching to the holoblastic mode, this structure may disappear, either because there is no longer a need for its determinative function, or because it may be dissolved into histologically not detectable molecular units throughout the isolated cell, and initiate molecular pathways, which may be involved in further specification of this cell and its derivatives.

A structure with the same characteristics is described during the first division cycles of the anomopod *Moina rectirostris*[52,53]. As shown for the **ncr**, it is composed of a spherical body of ca. 9 μm in diameter surrounded by dense granules forming a compact structure [52]. It disappears during the formation of the 16-cell stage. In this early study, it is considered to be a polar body (‘Richtungskörper’), but its origin has neither been investigated by [52] nor by [53]. In a histological study of *Daphnia pulex* Ojima [54] notes a cytoplasmic structure that ‘… contains a spherical body surrounded by small spherules…’ and is also found during the first cleavages. Von Baldass [48] mentions this structure in *D. pulex* as the vegetal pole plasm (original: ‘vegetatives Polplasma’) and Kaudewitz [55] refers to it in the description of the normogenesis for his centrifugation experiments. However, none of these authors gives a clear description about the structural identity of this structure in *D. pulex*[43,48,55].

Since the **ncr** is found in representatives of Onychopoda and Anomopoda, it would be interesting to clarify whether it is also found in Ctenopoda and Haplopoda. The study of *Holopedium gibberum* does not report such a structure [39]. In the superficially cleaving *Leptodora kindtii*, no structure comparable to the **ncr** has been described [50,51]. This is also true for the superficially cleaving *Simocephalus vetulus*, although the latter reveals a cell fate restriction of specific groups of cells at the blastoderm stage [49]. As a conclusion we assume the presence of an **ncr**-like structure during the early development as plesiomorphic for at least Onychopoda and Anomopoda. According to recent analyses of cladoceran phylogeny [41,56], this implies the occurrence of an **ncr**-like structure in the cladoceran stem species.

In the anomopod *D. magna*, the germ line during the early cleavage cycles has been visualized by using specific antibodies against the zinc-finger-containing Vasa protein [35]. Hence, this study supports the identity of the germ line precursor cell by a molecular marker. Nevertheless, a similar structure to the **ncr** has not been described [35]. The fixed position of such a marker within one specific cell may have special or different functions which should be subject to future functional studies. In this context it is interesting that eggs of *D. pulex* develop normal and viable hatchlings after the eggs have been centrifuged prior to the 1st division [55]. These eggs do not form the described cell pattern at the 16- or 32-cell stage, but nevertheless the emerging animals are not affected [55]. These facts and the different circumstances of the occurrence of the **ncr** in different cladocerans suggest that the stereotyped cell division pattern may not primarily be explained functionally, but rather point to a phylogenetic signal.

Structures comparable to the **ncr** have been described in some malacostracan embryos. For instance, a naturally occurring cell marker containing RNA has been found in the dendrobranchiate decapod *Penaeus monodon*[15]. The authors call this structure intracellular body (**icb**) and suggest that it marks the germ line of the species. A corresponding cytoplasmic marker has been found and has been morphologically characterized in other dendrobranchiate shrimps [57,58]. A recent study in the amphipod *Parhyale hawaiensis* characterizes a cytoplasmic germ cell marker via morphology and germ line associated RNAs [59]. The structure of the cytoplasmic marker in *P. hawaiensis* is similar to what is described as a cell marker in penaeid shrimps [15,58] and to the structure of the **ncr** of the present study. Interestingly, it could be shown that the cytoplasmic cell marker in *P. hawaiensis* plays a crucial role not only in the establishment of the identity of the germ line during early cleavages, but also in the proper initialization of the gastrulation [59]. Whether the presence of germ plasm as germ line determinant during the early cell lineage can be assumed for the tetraconate ground pattern requires additional functional studies on germ plasm in more crustacean groups and hexapods. However, cytoplasmic cell markers, which are mostly connected to the germ line, are frequently found among crustaceans, and it therefore seems likely to be a plesiomorphic feature for the Tetraconata.

### The transient appearance of cell morphological structures

In *Bythotrephes longimanus,* the occurrence of distinct granules is shown prior to the 1st division and due to the localization of the granules, it is concluded that eventually they aggregate around the **ncr**. Afterwards, similar granules could not be found in any later blastomere or cell of *B. longimanus*. This compact structure around the spherical part of the **ncr** does not change its appearance during following mitosis processes. Kühn [17] describes granules scattered over the cytoplasm in *Polyphemus pediculus* rather as a cyclic temporal appearance throughout the entire early development. These granules occur in each cell, but only when mitosis is completed and the nuclei enter the late telophase; then they enlarge and become more distinct. The granules disappear by the time of initiation of the following mitosis [17]. Therefore, in *P. pediculus* the structure associated with the **ncr** may not be formed by the dense cytoplasmic granules found in *B. longimanus*.

**Figure 9:**
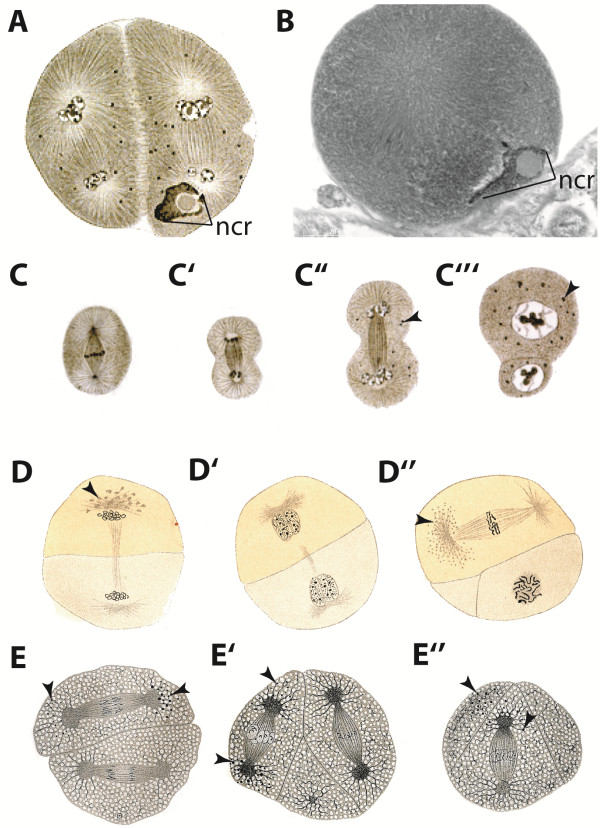
**Sub-cellular structures that can be observed temporarily during early development have been previously described in other crustaceans. A** The end of the 2nd division of *Polyphemus pediculus* after [17]. The **ncr** lies adjacent to the D nucleus. **B** The 2nd division in metaphase in *B. longimanus* with the **ncr. C’-C”’** The cyclic appearance of the granules during a cell cycle in *P. pediculus* after [17] exemplified by a mitosis of a cell at the late blastula stage. **D-D”** Granules in the copepod *Cyclops strenuus* during a cell cycle after [60]. **D** At the telophase of the first division the granules gathered around one pole of the mitotic spindle start to disappear and are not visible at the 2-cell stage in **D’. D”** During the next cycle the granules reappear at one pole during the initial metaphase. **E-E”** In *Cyclops viridis*[24] the granules are detected in more than only one mitotic pole (arrow heads in **E** and **E’**) or even in more than one cell during the first three divisions (arrow heads in **E”**).

Similar cytoplasmic granules are also found in copepods [24,60,61]. Here, the granules also appear periodically, but, in contrast to *P. pediculus*, they exclusively occur during the phase of mitosis [24,60,61]. Häcker [60] observes these granules in *Cyclops viridis* in one cell, i.e. in only one pole of one spindle, from the first cleavage on. He calls these granules ‘Ektosomen’ , a term that is later taken up by other authors [61,62]. They appear in the first cell which immigrates towards the center of the embryo and which gives rise to the germ line [60]. Fuchs [24], however, investigates the same species and detected these granules in more than one cell until the 3rd division cycle; he finds that it is not before the 4th division that the granules are restricted to one cell [24] (Figure 9).

The link between granules and germ line has been described for many other invertebrate groups (for review see [63]). In the rotifers *Asplanchna priodonta*[62] and *Asplanchna herrickii*[64], the cyclic occurrence of granules scattered around one pole of the mitotic spindle reveals an astounding similarity to what is described in copepods, leading Nachtwey [62] to apply Häcker’s [60] term ‘Ektosomen’. A more recent study using current techniques to visualize the architecture of the developing egg, however, does not mention the existence of granules in a bdelloid rotifer [65].

In conclusion, the function of cytoplasmic bodies as germ-cell determinants is suggested. However, there seems to be a more complex interaction between different histologically visible structures and the reported peculiarities in different species which may point to different functions within the germ line differentiation process. Therefore, in future studies it would be very interesting to study the spatial and temporal occurrence of cytoplasmic markers in more species.

Subdivided nuclei similar to the nucleus with its blister in *B. longimanus* have been described in sexually reproducing species such as the copepod *Cyclops* spec*.*[60] and in the marine snail *Crepidula* spec. [66]. In these cases the nuclei appear divided into two equally sized compartments based on an incomplete fusion of the female and the male gamete. Even during the subsequent division cycles the nuclei always rearrange to the two separate partitions [60,66]. The same is reported for the fertilized eggs of the rotifer *Asplanchna intermedia* and even for parthenogenetic eggs [67]. Interestingly, in the parthenogenetic eggs of *Artemia salina* the maturation division can give rise to either only one polar body or even two [18,68]. In the latter case, the second polar body reunites with the egg nucleus [18]. If a similar process is assumed for *B. longimanus*, the occurrence of the nuclear blister may be explained. However, with the involvement in the spindle during the 1st mitosis it should then also be assumed that parts of the fused nucleus are separated again during the first mitosis.

### The ancestral cleavage pattern of Cladocera

Studies of the early embryonic development with respect to cell lineage and cleavage pattern are known from representatives of all four major groups of cladocerans (Onychopoda, Anomopoda, Ctenopoda and Haplopoda (i.e. *Leptodora kindtii*)). The monophyly of these groups is generally accepted. However, with respect to the relationship among these taxa there are two hypotheses basically concerning the position of *L. kindtii*: according to [69]*L. kindtii* branches basally as the sister group to the remaining Eucladocera, reflecting the classical textbook view of the taxonomical system of Cladocera [70]. Within the Eucladocera, the Onychopoda form the sister group to the monophylum comprising Ctenopoda and Anomopoda [69]. According to the second hypothesis, *L. kindtii* is the sister group to the Onychopoda, both together forming the Gymnomera [56,71]. The latter hypothesis is well supported by a combined analysis of morphological and molecular data [41]. Whether the Anomopoda or the Ctenopoda are more closely related to the Gymnomera remains controversial [72]. Nevertheless, some analyses speak in favor of the Ctenopods as the sister group to the Gymnomera [41,56].

Similarities in the early development of cladocerans have been previously described [4,17,32,48,73]. However, most comparisons are linked to the presumed cell fate determination through gastrulation [3,32]. Here we focus on the cleavage pattern with respect to the orientation and sequence of early mitotic divisions. Inspecting the cell patterns that are formed during the earliest cell divisions, the following correspondences can be stated for representatives of Ctenopoda, Onychopoda, and Anomopoda:

I. The first two divisions are adequal and meridional. In those cases in which the eggs form blastomeres, irrespective of whether or not the membranes completely penetrate the egg, cross furrows between the two opposed non-sister cells are formed at right angles to each other, as e.g. in *Holopedium gibberum*. At one pole (designated as vegetal pole) it is the smallest **D** derivative that is in contact to **B** forming the **BD** cross furrow and at the other pole (designated as animal pole) it is **A** that contacts **C** as the **AC** cross furrow. The cross furrows can be less obvious, as shown for *Bythotrephes longimanus*; however, it is never **A** that is in contact to **C** at the animal pole during the 4-nuclei stage. The cross furrow becomes more pronounced at the 8-cell stage. The same is true for *Moina rectirostris*. Here, additional contact planes between micro and macromeres that are not direct sister cells are found as described also in *P. pediculus*. In *B. longimanus*, this observation is not clearly confirmed since some variations with respect to the cell boundaries occur.

**Figure 10:**
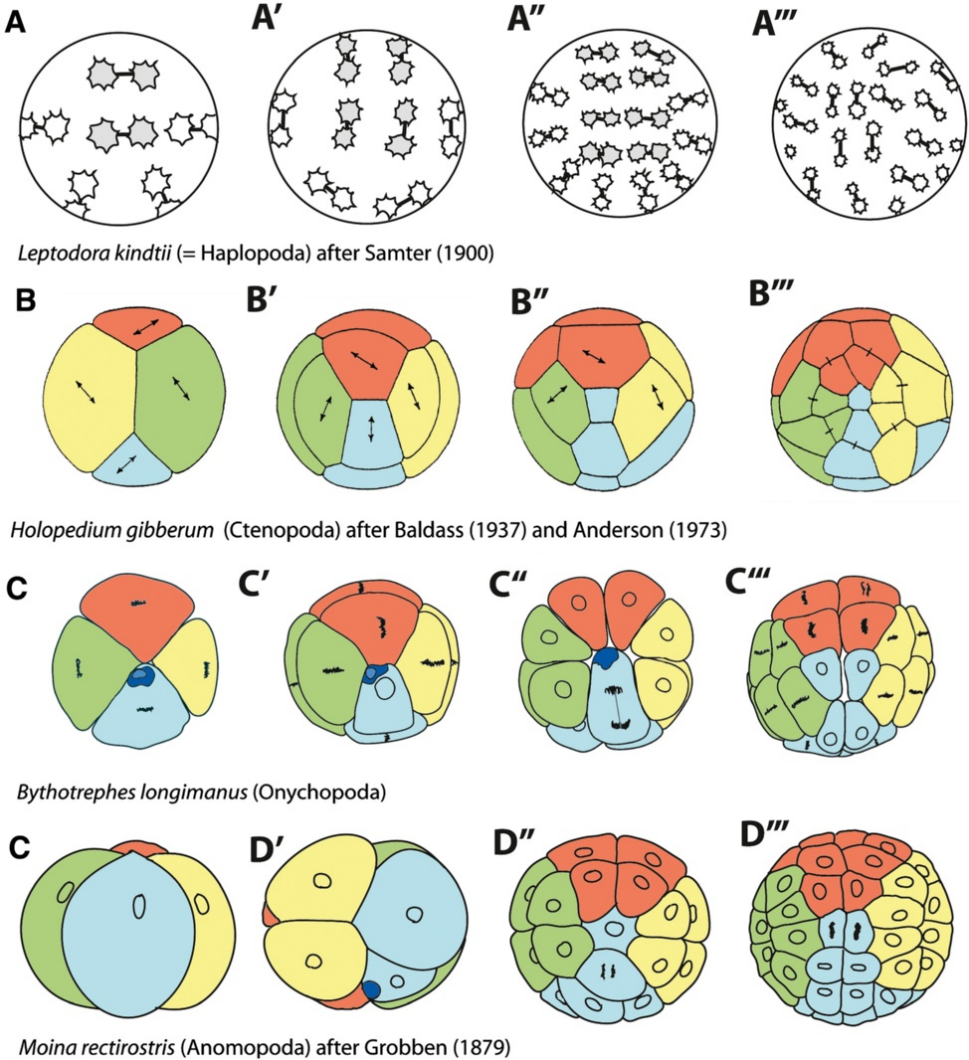
**Schematic comparison of representatives of the four major cladoceran groups.** The coloration and the orientation are adapted according to the quadrants of the 4-cell stage in Figures 7 and 8 in order to simplify the comparison, with A - yellow, B - red, C - green, D - blue. Dark blue - **ncr**. Connecting lines indicate sister cells, double arrows indicate the direction of divisions during the subsequent division. **A-A”’** The four consecutive division cycles of *Leptodora kindtii* starting with the fourth division in **A** (after [50]). The cell lineage was not traced in [50], however, the pattern of the two interlocking cell bands is indicated here by gray versus white filled energids. **B-B”’** The four consecutive division cycles of *Holopedium gibberum* after [3,39] starting with the third division in **B. B** View of the animal pole presenting the AC cross furrow. **B’**-**B”’** View of the vegetal pole. **C**-**C”’** The four consecutive division cycles of *Bythotrephes longimanus* starting with the third division in **C**, all views of the vegetal pole. **D-D’”** The four consecutive division cycles of *Moina rectirostris* after [52] starting with the 4-nuclei stage in **D. D** Lateral view of the D quadrant with the animal pole facing down. **D’** Lateral view of the A and D daughter cells with the animal pole facing up. **D”**-**D”’** View of the vegetal pole.

II. The delay in division of one **D** derivative as the assumed presumptive primordial germ cell is apparent in all the representatives depicted in Figure 10 (except *L. kindtii*). This delay is not described for *Daphnella* species [53] or *Simocephalus vetulus*[49], which may be due to the difficulty in tracing cell lineage pattern in yolky superficially cleaving eggs. The division from which the primordial germ cell emerges (**gc** in *P. pediculus* and *B. longimanus*) deviates from the divisions of the other cells, since it occurs at a right angle to the **BD** cross furrow. With which division cycle this happens is not fixed within the different groups. For instance, in *H. gibberum* and *B. longimanus* this deviant and cell fate determining division occurs during the 4th cycle; in *M. rectirostris* it is one cycle later. Regardless of the timing, this division pattern leads to a characteristic cell arrangement at the vegetal pole, in which the contact plane of the former **BD** cross furrow is still preserved between **B** and **D** derivatives and the **A** and **C** derivatives therefore do not contact each other at this pole.

Depending on which hypothesis is taken as a basis for the phylogenetic relationships between the four cladoceran taxa, the described cleavage pattern for representatives of Anomopoda, Ctenopoda, and Onychopoda can be interpreted differently. In the case of Haplopoda as the sister group of all other cladocerans, this pattern may have evolved within the cladocerans in the stem species of the Eucladocera. One argument in favor of this hypothesis could be the pattern of two interlocking cell bands in *L. kindtii* that appear similar to what is found in malacostracans like Dendrobranchiata and Euphausiacea [12,74] (but see below). However, assuming the monophyly of the Gymnomera with the two raptorial cladoceran groups, Onychopoda and *L. kindtii*, being sister groups, the cell division pattern of *L. kindtii* must be derived. As a consequence, the described pattern for representatives of Anomopoda, Ctenopoda, and Onychopoda may have existed in the stem species of the Cladocera or may even represent a plesiomorphic state for a larger group within Branchiopoda.

### Comparison of cleavage patterns in non-malacostracans and malacostracans

The traditional view splitting Crustacea into Entomostraca and Malacostraca is not well supported by phylogenetic analyses. Only Walossek [75] found some evidence for monophyletic entomostracans, but the majority of analyses suggested that entomostracans are a paraphyletic or even polyphyletic group [5-7,76]. Nevertheless, starting from the older literature on comparisons of crustacean development [39,48], embryologists have consistently compared these two groups until recently [32]. In a review of cell lineage and germ layer formation during gastrulation in Crustacea, Gerberding and Patel [32] stress the differences between Malacostraca and the remaining crustaceans and do not find shared characters with respect to the pattern of germ layer formation. Other comparative studies, in contrast, emphasize the similarities in the early cell lineage pattern of holoblastically cleaving malacostracans and non-malacostracan taxa [2,3].

Within malacostracans, invariant patterns are described in dendrobranchiates within decapods [13-15,77,78], in euphausiids [11,12], and in amphipods [8-10]. The distinct similarities found in the early cleavage pattern of dendrobranchiates and euphausiaceans have led to the assumption that these patterns are homologous [12,14] but not necessarily synapomorphic for the two taxa [74]. One of the identified correspondences concerns the arrangement of the blastomeres at the 4-cell stage with two non-sister cells forming a cross furrow at opposing poles. These cross furrows are perpendicular to each other resulting into two interlocking cell bands during the following division cycles in which the mitotic spindles orient in the same direction. Apart from other decapods (see figures in [79]), a cleavage pattern of two interlocking cell bands has not been described for other malacostracan groups, neither holoblastic nor superficially cleaving malacostracans (see e.g. [10,80,81]).

There are some examples of non-malacostracans that apparently reveal a pattern of two cell bands that are arranged in a similar way as described for decapods and euphausiaceans. In the ostracod *Cypris incongruens*, the arrangement of the blastomeres forming two cross furrows at opposing poles leads to the formation of two interlocking cell bands in which the spindles alternately orient end to end and parallel to each other [73]. As a consequence, the cross furrows of the 4-cell stage are preserved and broadened during the following cleavages. In copepods, a pattern similar to the interlocking cell bands is not described in the text, but following the figures of histological sections with a parallel orientation of groups of cells may indicate such a pattern [24]. The Copepoda display many holoblastically cleaving examples reaching from nearly equal to clearly unequal [24,60,82,83]. Interestingly, some parasitic copepods reveal a cell arrangement during gastrulation [82] that reminds of what is found in euphausiids, which has also been noted by [11]. Indications for two interlocking cell bands for the cladoceran *Leptodora kindtii* have already been mentioned above. The genealogical composition of these cell bands is not clear from what has been described [50].

A pattern of an interlocking cell band in the remaining cladocerans has not been described before [17,39,48,52] and such a pattern is not clearly apparent in *Bythotrephes longimanus* either. The blastomeres and their derivatives that could potentially form a cell band, like e.g. **A** and **C** and their derivatives, do not show a particular regular division behavior as would be expected for a cell band pattern. However, we want to stress here that the contact planes between **A** and **C** blastomeres at the animal pole and **B** and **D** blastomeres at the vegetal pole, respectively, are preserved until later stages. Consequently, **B** and **D** derivatives at the animal pole and **A** and **C** derivatives at the vegetal pole never contact each other.

The knowledge about details of holoblastic or mixed cleavage in hexapods is scarce. Plesiomorphically, these cleavage modes occur in Collembola, Protura, and Machilidae [84-87]. Some figures in [84] and [85] suggest that there is a blastomere arrangement in the 4-cell stage that is similar to what is seen in cladocerans such as *B. longimanus*. However, to confirm this, more detailed investigations of hexapod total cleavages are necessary. It is evident that the ancestral cleavage of Tetraconata was holoblastic (see [1]). Here, we suggest that the formation of a blastomere distribution in which the non-sister cells at the 4-cell stage are in contact at one pole and not at the opposite pole could additionally provide the common plesiomorphic pattern of the tetraconate early cleavage pattern.

## Conclusions

In this study we provide a detailed description of the early development and cell lineage onychopod cladoceran *Bythotrephes longimanus* using recent methods. We can show that the early development of *B. longimanus* is very similar to that of the polyphemid onychopod *Polyphemus pediculus.* This indicates that this mode of development is part of the ground pattern of the Onychopoda. The comparison to the cleavage patterns of other cladocerans allows the reconstruction the original cleavage pattern of Cladocera as a whole, or at least of the last common ancestor of the three major cladoceran taxa Anomopoda, Ctenopoda and Onychopoda: (1) the first two cleavages are adequal and meridional, irrespectively whether the cleavages are complete or partial, (2) one blastomere or quadrant resulting from the second cleavage is smaller and division-delayed during the subsequent divisions, originally containing a morphological cell marker which likely gives rise to the germ line, and (3) a clonal pattern during the subsequent divisions is formed, in which the cells deriving from the none-sister cells at the 4-cell stage preserve a shared contact zone. We suggest that a clonal distribution, in which the clones of none sister cells at the 4-cell stage remain in contact at one pole and not at the other pole during the subsequent cleavage cycles, could be described as a common plesiomorphic pattern of tetraconate early cleavage pattern.

## Materials and methods

### Animals and embryos

Specimens of *Bythotrephes longimanus* were collected during the parthenogenetic reproductive season in the summer of 2006 at the Tegeler See in Berlin (Germany). Samples were taken at a depth of 0.5 m with a plankton net (HYDRO-BIOS, mesh size 100 μm) and kept in cooled water containers (2–5 l) for transport. *B. longimanus* females revealing dorsal brood pouches with embryos about to hatch were isolated in filtered fresh lake water and kept at 15°C. The females shed the brood pouch with the hatching juveniles and the newly formed brood pouch containing the early stage eggs immediately occurred as a small triangular lobe (Figure 1).

### 4D-microscopy and cell lineage analysis

The principles and general components of the 4D-microscopy system as a multiple focal plane time-lapse recording system are described in detail by [88,89]. The system in this study was composed of a motorized Zeiss Axiophot (Axioplan II Imaging) equipped with a PCO pixelfly camera. The embryos were recorded using the C-Apochromat 63x/1.2 W objective (Zeiss). The cell lineage was analyzed with SIMI°BioCell 4.0.153 (SIMI° Reality Motion Systems GmbH, Unterschleissheim, Germany). The development of the embryos of *B. longimanus* is dependent on the conditions of the brood pouch of the adult female, and opening the dorsal brood pouch immediately damaged the embryos. Therefore, the adult female with its brood pouch containing the early stages was put into a flow-through chamber using cooled filtered lake water. The freshwater flow-through chamber was connected to a diaphragm metering pump (STEPDOS® 03 S, KNF Neuberger GmbH, Germany), which pumped oxygenated filtered freshwater (cooled to 10°C) with 0.133 ml/s through the chamber. As the pulsing of the diaphragm metering pump interfered with the recording, the pump was only turned on between scans. The advantage of the movement of the cover slip was that the pumping rhythm had an effect similar to a ‘pacemaker’ , because the movement seemed to reactivate the heartbeat. This extended the length of life of the adult females by at least two hours.

### Fluorescence staining and analysis

Relevant stages were fixed in 3.7% formalin-PBS for 15 to 30 min. After fixation, the eggs were rinsed in PBS and transferred to absolute methanol for storage at −8°C. Following washing steps in PBS the embryos were stained in either Hoechst (H33258, Molecular Probes) for 15 min or in Sytox®Green (Molecular Probes) for about 3 hours. The embryos were mounted in DABCO-glycerol and analyzed with a Zeiss Axioskop II and Leica TCS SP2 AOBS. The image stacks of the confocal laser scanning microscopy were further analyzed using the 3D image visualization software Imaris 5.0.3. (Bitplane AG, Zürich).

### Histology and auto-aligning

For semi-thin sectioning, embryos fixed in Bouin’s fixative were washed in PBS several times. In order to facilitate further handling and orientation of the embryos, they were gently pre-treated with 10% Delafield’s haematoxylin solution (Merck). Following dehydration in a graded ethanol series, the embryos were transferred to a metacrylate embedding medium (Technovit, Kulzer) as per the company’s instruction. Serial semi-thin sections (1–3 μm) were obtained by a motorized rotary microtome (MICROM HM 355). Dried sections were stained with either 0.5% toluidine blue or methylene-azur/fuchsin following standard protocols and embedded in Histokitt (Roth). The sections were digitized using an AxioCam HRc (Zeiss) connected to an Axioskop II (Zeiss). For 3D visualization, images were converted to gray scale, adjusted in levels and contrast, and handled with the un-sharp mask (50%) using Adobe Photoshop 7.0. The alignment of the picture series was done with Autoaligner 2.0 (Bitplane AG, Zurich).

### Nomenclature

The nomenclature is based on the four capital letters **A**, **B**, **C**, and **D** that emerge from the two nuclei **AB** and **CD**. At the 8-nuclei stage, the smaller ‘blastomeres’ (referred to as micromeres *sensu lato*, although they are not separate cells or blastomeres) are designated with lower case letters **aII**, **bI**, **cII**, and **dI**, and the larger ones (denoted as macromeres *sensu lato* as above) with the capitals **AI**, **BII**, **CI**, and **DII**. The indices I and II are used in order to additionally distinguish between the cells of opposing quadrants that are in contact by the addition of indices I, i.e. **bI** and **dI**, which are in contact at the vegetal pole and **AI** and **CI,** which are in contact at the animal pole. In the following division cycles, their derivatives are labeled counterclockwise in view of the vegetal pole by the addition of ‘1’ or ‘2’ , e.g. the micromere **aII** at the 8-nuclei stage is divided into **aII1** and **aII2** during the 4th division cycle to the 16-cell stage. Following the nomenclature of [17], the daughter cells of the **D** micromere at the 16-cell stage are designated **gc** and **en**, with **gc** being the cell adjacent to the **B** derivative cells and containing the morphological marker **ncr**, and **en** being its sister cell. The abbreviations **gc** and **en** refer to translations of Kühn [17] and correspond to ‘Kz’ (gc), used by Kühn [17] as an abbreviation for the German term ‘Keimzelle’ (germ cell) and to ‘en’ (en) for the German term ‘Entodermzelle’ (endoderm cell). Subsequent generations of cells are continuously marked by ‘1’ or ‘2’ as indices as above.

## Competing interests

The authors declare that they have no competing interests.

## Authors’ contributions

FA proposed the idea for the research, designed and performed the experiments, analyzed data, and wrote the manuscript. GS discussed the various research steps with FA, revised the manuscript and obtained funding for the research. Both authors read and approved the final manuscript.
